# Yellow fever in Peru and the Americas and the latent risk of reurbanuzation: an avoidable threat

**DOI:** 10.17843/rpmesp.2025.422.15114

**Published:** 2025-06-20

**Authors:** César Cabezas

**Affiliations:** 1 Instituto Nacional de Salud. Lima, Peru. Instituto Nacional de Salud Lima Peru

**Figure 1:**
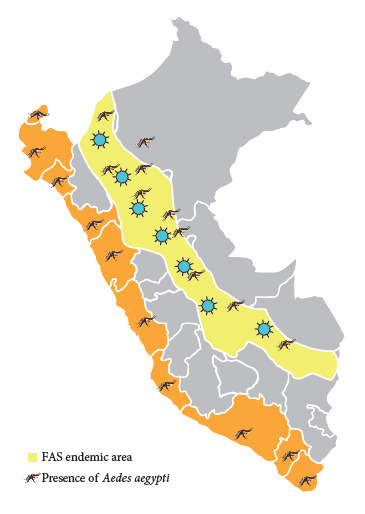
Distribution of Aedes aegypti in Peru.

For decades, urban yellow fever (UYF) was one of the most feared diseases in most areas of the Americas. Since its urban eradication in the first half of the 20th century,achieved through the elimination of *Aedes aegypti,*^1^^,^^2^ the virus has remained restrained to the sylvatic yellow fever (SYF) cycle, transmitted by wild vectors in the Amazon Basin. As of 2025, a total of 235 cases and 96 deaths have been reported in South America ^3^, including 42 cases and 16 deaths in Peru until June 14th ^4^; this reflects the re-emergent trend of the disease in the region despite the efforts for achieving control in these countries. In this context, some converging factors have highlighted the risk of yellow fever re-urbanization in the Americas, particularly in South America, where ecological, social, and health-related conditions create a favorable environment for this resurgence. The growing presence of the urban vector *Aedes aegypti* in densely populated cities and even in small towns located near forests where sylvatic yellow fever (SYF) cases are currently occurring is one of the main factors (Figure 1). This risk is further exacerbated by the progressive urban adaptation of traditionally sylvatic vectors such as some species of *Haemagogus* and *Sabethes*^5^, driven by deforestation and the human incursion into their ecological niches. Of even greater concern is the low vaccination coverage, both in endemic regions as well as in non-Amazonian urban areas, as well as the intense human mobility of unvaccinated population to and from endemic areas due to economic and social reasons. All of these factors are compounded by overarching determinants such as climate change and unplanned urbanization.

Studies such as that by Massad *et al.*^6^ provide a valuable tool for quantifying this risk through a mathematical model, the conclusions of which are cause for concern. According to the model, in cities heavily infested with *Aedes aegypti*, a single infected traveler returning from a yellow fever endemic area can be enough to trigger an urban transmission chain if the vectorial competence of the mosquito exceeds 70% relative to that observed for dengue.

Although this threshold has not yet been reached in most South American *Aedes aegypti* strains, recent ecological changes—such as the displacement of *Haemagogus* mosquitoes into peri-urban areas, the low vaccination coverage outside of Amazonian regions and the increased human mobility, suggest that this threshold is dangerously close to being met and surpassed.

Moreover, recent outbreaks of SYF in non-Amazonian regions of Brazil and Colombia, along with the expansion of *Aedes aegypti* into coastal and mid-altitude areas, should be interpreted as warning signals ^7^.

The potential for harm is massive: high mortality, the possible collapse of urban health systems, risk of international spread (including to Asia), and a critical shortage of vaccines, the production of which is limited to no more than four WHO-prequalified centers worldwide ^8^. This underscores the urgent need not only to strengthen prevention and control measures, but also to strategically prepare health services to manage cases across all levels of care—from primary to tertiary—through the systematic training of the health workforce.

It is important to emphasize that yellow fever is a disease that can be fully prevented through vaccination. However, this requires ensuring adequate and timely production and supply of vaccines, particularly in endemic countries facing increasing risk. Epidemiological surveillance must be expanded to include syndromic surveillance, as the early clinical presentation of yellow fever can be easily mistaken for other arboviral infections such as dengue, Zika, chikungunya, Mayaro, or Oropouche, or other infections such as malaria or leptospirosis, depending on the local epidemiology. This implies the availability of rapid immunochromatographic tests for the detection of viral antigens and/or specific antibodies against yellow fever, which should be offered at the basic health care level—in particular in rural and peri-urban populations of the Amazon. Alternatively, accessible multiplex molecular testing platforms should be implemented in these areas.

All of the above highlights the pressing need for countries in the Global South to advance towards their technological autonomy in the diagnosis, prevention, and response to neglected infectious diseases, such as yellow fever and other arboviral infections. As these diseases do not represent profitable markets, they are not prioritized by major transnational pharmaceutical companies, so regional health sovereignty is essential to ensure timely and effective responses.

Facing this situation, it is urgent to adopt a proactive approach that should include: a) A significant improvement in yellow fever vaccination coverage within the Expanded Program on Immunization (EPI) for children, as agreed upon by countries in the Americas since 1998. This must prioritize at risk rural and peri-urban areas, with the strategic use of fractional dosing of available vaccines during outbreaks in the event of limited supply; b) Strengthening epidemiological and entomological surveillance, as well as enhanced syndromic and genomic diagnostic capabilities. This involves monitoring the presence of sylvatic vectors in Amazonian populations and analyzing their role in yellow fever virus transmission, as well as intensifying *Aedes aegypti* vector control through integrated community-based and technological interventions, in particular in small towns located near SYF endemic zones—without relaxing the control of this vector in regions affected by dengue; c) Promoting risk communication and education campaigns engaging local stakeholders, incorporating intercultural approaches tailored to migrant populations moving from the Andes to the Amazon; d) Applying a One Health approach with multisectoral and community participation, linking surveillance of non-human primates (epizootics), humans, and vectors in forest-urban interface areas; and e) Strengthening vaccine production capacity—including investment in alternative technologies (such as recombinant, mRNA, or inactivated platforms) to enable larger-scale, safe, and effective vaccine manufacturing—alongside the development of rapid diagnostic tests in the region. This must be accompanied by collective efforts among countries of the Global South to reduce external technological dependence, which was starkly revealed during the COVID-19 pandemic. Achieving these objectives will require renewed solidarity among nations and timely political decision-making in order to tackle shared threats.

The return of urban yellow fever and the occurrence of sylvatic yellow fever outbreaks are preventable—even in the current context—provided that action is taken in a timely fashion rather than reactively. Science and technology have already provided effective tools to estimate and anticipate the risk. It is now the responsibility of health systems, political authorities, and communities to implement coordinated and immediate responses to avoid repeating the history in the region.

*Yellow fever can re-emerge in urban settings. The real question is not whether it will happen, but whether we will allow it to happen.*
