# Supplementary material for: Yellow fever in Peru and the Americas and the latent risk of reurbanuzation: an avoidable threat
Source: Rev Peru Med Exp Salud Publica. 2025 Jun 20;42(2):113–4. doi: 10.17843/rpmesp.2025.422.15114 (PMC12377879; doi:10.17843/rpmesp.2025.422.15114)
Supplement: Translation

# Translation: Fiebre amarilla en el Perú y las américas, el riesgo latente de su reurbanización: una amenaza evitable

**Authors:** César Cabezas

**Affiliations:** 1 Instituto Nacional de Salud. Lima, Perú.

Durante décadas, la fiebre amarilla urbana (FAU) fue una de las enfermedades más temidas casi en todo en el continente americano. Desde su erradicación urbana en la primera mitad del siglo XX al eliminarse la presencia del *Aedes aegypti*^1^^,^^2^, el virus se ha mantenido limitado al ciclo de la fiebre amarilla selvática (FAS), transmitido por vectores silvestres en la Amazonia. En lo que va del 2025 se han presentado 235 casos con 96 fallecidos en Sudamérica ^(^^3^ y al 14 de junio ocurrieron 42 casos con 16 fallecidos en el Perú ^4^, manteniendo su tendencia reemergente en la Región, pese a los esfuerzos de control en los países. En este contexto, un conjunto de factores convergentes ha puesto nuevamente en evidencia el riesgo de reurbanización de la fiebre amarilla en el continente, en especial en Sudamérica, donde las condiciones ecológicas, sociales y sanitarias crean un escenario propicio para su resurgimiento urbano, como es la presencia cada vez mayor del vector urbano *Aedes aegypti* en ciudades densamente pobladas y aun en pequeños poblados cercanos a la floresta donde actualmente se dan los casos de FAS (Figura 1), agregado a la urbanización progresiva de vectores silvestres como algunas especies de *Haemagogus* y *Sabethes*^5^ debido a la deforestación y la invasión de sus nichos ecológicos por la actividad humana, más serio aún es la baja cobertura vacunal en general en zonas endémicas y en zonas urbanas no amazónicas, también la intensa movilidad humana hacia y desde zonas endémicas por razones económicas y sociales de población no vacunada. Todo lo anterior en medio de determinantes mayores como son el cambio climático y la urbanización desordenada.

**Figura 1:** Dispersión de *Aedes aegypti* en el Perú.

Hay estudios como el de Massad *et al.*^6^ que proporciona una herramienta para cuantificar este riesgo, a través de un modelo matemático cuya conclusión es preocupante porque en ciudades densamente infestadas por *Aedes aegypti*, un solo viajero infectado que regrese desde una zona endémica de fiebre amarilla puede bastar para detonar una cadena de transmisión urbana si la competencia vectorial del mosquito supera un umbral del 70% comparado al dengue.

Aunque ese umbral aún no ha sido alcanzado en la mayoría de cepas sudamericanas de *Aedes aegypti*, los cambios ecológicos recientes -como el desplazamiento de *Haemagogus* hacia áreas periurbanas-, la baja cobertura vacunal fuera de zonas amazónicas y el aumento de la movilidad humana, hacen que este umbral esté peligrosamente cerca de ser superado.

Más aún, los brotes recientes de FAS en zonas no amazónicas de Brasil y Colombia, junto con la expansión del *Aedes aegypti* a regiones costeras y de altura intermedia, deben ser entendidos como señales de advertencia ^7^.

El potencial de daño es inmenso: alta mortalidad, colapso de sistemas de salud urbanos, riesgo de diseminación internacional (incluyendo Asia), y una escasez crítica de vacunas, cuya producción está concentrada en no más de cuatro centros en el mundo registrados por la OMS ^8^. Esto implica que paralelamente a reforzar las medidas de prevención y control, preparar estratégicamente los servicios de salud para atender pacientes desde el primer nivel de atención hasta los de mayor complejidad, incluyendo la sistemática capacitación de los recursos humanos de salud.

Cabe resaltar que la fiebre amarilla es una enfermedad totalmente prevenible mediante la vacunación. Sin embargo, ello requiere garantizar una producción y abastecimiento adecuado y oportuno, especialmente en países endémicos que enfrentan riesgos crecientes. La vigilancia epidemiológica debe ampliarse para incluir la vigilancia sindrómica, dado que los cuadros iniciales de FA pueden confundirse con otros arbovirus como dengue, Zika, chikungunya, Mayaro, Oropouche, Malaria, Leptospira u otros según el perfil epidemiológico. Esto exige el uso de pruebas rápidas inmunocromatográficas para la detección del antígeno viral y/o anticuerpos específicos contra FA, que deben estar disponibles en el primer nivel de atención, sobre todo en áreas rurales y periurbanas en poblaciones de la Amazonia, alternativamente plataformas multiplex de pruebas moleculares que sean accesibles en estas zonas.

Todo lo anterior pone de manifiesto la necesidad -casi urgente- de que los países del hemisferio sur avancen hacia una autonomía tecnológica en el diagnóstico, prevención y respuesta ante enfermedades infecciosas desatendidas como es el caso de la fiebre amarilla y otros arbovirus. Estas patologías, al no representar grandes mercados, no son prioritarias para las grandes compañías transnacionales, por lo que la soberanía sanitaria regional resulta esencial para tener una respuesta oportuna.

Frente a este panorama, se hace urgente adoptar un enfoque proactivo, que deba considerar: a) Una sustancial mejora de las coberturas de vacunación antiamarílica dentro del programa ampliado de inmunizaciones en niños, como fue el acuerdo de los países de las Américas desde 1998, también priorizando zonas rurales y periurbanas en riesgo, con uso estratégico de dosis fraccionadas durante brotes si no hubiera suficiente número de vacunas, b) Fortalecer la vigilancia epidemiológica y entomológica, incluyendo capacidades de diagnóstico sindrómico y genómico. En particular vigilar la presencia de vectores rurales en poblaciones de la Amazonia que incluya la incriminación de estos vectores con el virus de FA, igualmente intensificar el control vectorial de *Aedes aegypti*, integrando medidas comunitarias y tecnológicas sobre todo en localidades cercanas a áreas endémicas de FAS, sin dejar de hacer el control de este vector en otras regiones con dengue. c) Impulsar campañas de educación y comunicación del riesgo, que involucren a actores locales, tiendo en cuenta un enfoque intercultural en poblaciones migrantes de los andes a la amazonia, d) Aplicar un enfoque One Health con participación multisectorial y comunitaria, que articule la vigilancia de primates (epizootias), humanos y vectores en áreas de interfaz selvática-urbana, finalmente e) Fortalecer capacidades de producción de vacunas - buscando también alternativas tecnológicas (recombinantes, mRNA o inactivadas) para una producción de vacunas en mayor proporción-que también sean seguras y eficaces-, así como pruebas diagnósticas rápidas en la Región, sumando esfuerzos de los países del hemisferio Sur para reducir la dependencia tecnológica externa, como la experimentamos en la última pandemia del COVID-19. Para esto se requiere enfatizar la solidaridad entre nuestros países y las decisiones políticas oportunas frente a amenazas comunes.

El regreso de la fiebre amarilla urbana y la ocurrencia de brotes de la fiebre amarilla selvática son evitables, incluso en el contexto actual, si se actúa con oportunidad y no de forma reactiva. La ciencia y la tecnología ya han puesto a nuestra disposición herramientas eficaces para estimar y anticipar el riesgo. Corresponde ahora a los sistemas de salud, a las autoridades políticas y a las comunidades implementar respuestas articuladas y urgentes para no repetir la historia en la Región.

*La fiebre amarilla puede resurgir en los entornos urbanos. La verdadera pregunta no es si ocurrirá, sino si permitiremos que ello ocurra.*
